# Protective effect of green tea extract on the deltamethrin-induced toxicity in mice testis: An experimental study

**DOI:** 10.18502/ijrm.v17i5.4601

**Published:** 2019-06-26

**Authors:** Hoda Bagherpour, Abbasali Karimpour Malekshah, Fereshteh Talebpour Amiri, Mohammad Azadbakht

**Affiliations:** ^1^Department of Anatomy, Faculty of Medicine, Molecular and Cell Biology Research Center, Mazandaran University of Medical Sciences, Sari, Iran.; ^2^Student Research Committee, Faculty of Medicine, Mazandaran University of Medical Sciences, Sari, Iran.; ^3^Department of Pharmacognosy, Faculty of Pharmacy, Mazandaran University of Medical Sciences, Sari, Iran.

**Keywords:** Deltamethrin, Green tea, Oxidative stress, Testis, Caspase-3.

## Abstract

**Background:**

Deltamethrin (DM) is one of the environmental factors that can have destructive effects on the male fertility. Green tea (GT) as a medicinal herb, has antioxidant property.

**Objective:**

The present study investigated the protective role of GT extract in improving the harmful effects of DM on the testis.

**Materials and Methods:**

In this experimental study, 35 adult male mice (25–30 gr) were divided in to five groups (*n* = 7/each). The control group received only normal saline. Sham received 0.2 ml corn oil. Green tea group received only GT of 150 mg/kg. bw; deltamethrin group received the DM at a dose of 0.6 mg/kg. bw; GT + DM received both GT and DM. The effect of GT was assessed by measuring oxidative stress markers, sperm parameters, histological and immunohistochemical analysis.

**Results:**

The results showed that the count and motility of spermatozoa, testosterone, and Malondialdehyde significantly decreased (p < 0.001) and the abnormal spermatozoa increased (p < 0.001) in DM group compared to control group. Moreover, enhanced caspase-3expression and apoptosis were observed in DM-treated mice compared to control group. Histologically, DM with a degenerative effect on testicular tissue reduced the spermatogenesis progenitor cells. The epithelial height and the diameter of the seminiferous tubules were also reduced in the DM group. Treatment with GT in the DM-treated mice significantly improved these changes.

**Conclusion:**

With these findings, it was concluded that the GT treatment with antioxidant activity and anti-apoptotic property could protect the testicular injury induced by DM.

## 1. Introduction

Deltamethrin (DM) [(1R,3S) [α-cyano (3-phenoxyphenyl) methyl]-3-(2,2-dibromo-ethenyl)-2, 2-dimethyl-cyclopropanecarboxylate] is a type-II pyrethroid synthetic insecticide that is widely used in agriculture, horticulture, forestry and veterinary medicine to maintain public health (1, 2). One of the environmental factors that affect the testicular tissue is DM that damages testicles and ultimately affects the fertility in men (3). Due to its high bioavailability at low concentrations, high resistance to light and low toxicity in mammals and birds, this agent is widely used (4). DM causes toxicity in various organs such as gonads by producing reactive oxygen species and increasing free radicals and producing oxidative stress and lipid peroxidation, as well as reducing the antioxidant enzymes activities and apoptosis, and thus adversely affecting the fertility (1, 5, 6). Several studies have examined the toxic effects of DM on the testicles (7, 8).

Natural antioxidants, like some compounds in medicinal plants, can play anessential role in reducing the harmful effects of free oxygen radicals on testicles and act as oxidative stress inhibitors (4). One of the most important edible herbs that contains antioxidant compounds is green tea (GT) (1, 9). Green tea leaves largelycontain polyphenolic flavonoids in which the main components are catechins. The essential catechins in the GT are Epicatechin, Epigallocatechin, Epicatechin gallate, and Epigallocatechin gallate (1, 10). Green tea has been proven to have many biological properties, including antioxidant, anticarcinogenic, anti-inflammatory, antimicrobial, and antimutagenic effects (11). Although, the exact mechanism of action of GT polyphenols is unclear (10), over the past decade, GT has been shown to have high antioxidant properties and high potency in the elimination of free oxygen radicals and trapping free hydroxyl and anion superoxide radicals, as well as anti-inflammatory, anticancer, and antimicrobial properties have attracted much attention (9, 10).

Based on our knowledge, no study has been recorded to investigate the simultaneous use of DM with GT and its effect on testicular changes. Hence, this study was conducted to evaluate the biochemical, histochemical, histopathological, and immunohistochemical changes induced by DM in testicular tissue and the protective effects of GT against these impairments.

## 2. Materials and Methods

### Green tea leaf extraction and HPLC analysis

Green tea leaves were gathered from the Ramsar GT field and their herbarium samples were prepared (Voucher number: E1-242-11). The leaves were then dried in the shade at room temperature. For extraction, dried leaves were crushed in a 355 mesh particle size. Then, 15 gr of GT leaf powder was added to 1000 ml of boiling water and stirred for 15 min on a shaker. It was allowed to cool at room temperature. The extract was filtered using a filter paper and stored in the refrigerator. The extract was freshly prepared for use every time. For the standardization of the extract, the amount of water-soluble extractive, total phenol, and flavonoid content were measured. Finally, the dose of 150 mg/kg of dried powder was dissolved in normal saline and administered to mice by gavage.

Folin-Ciocalteu phenol reagent (Merk, NO: 1/09001, Germany) was used as a marker and Gallic acid (Merk; NO: 842648, Germany) as the required phenolic standard for assay. The Folin-Ciocalteu reagent is susceptible to reductive compounds such as phenols the reaction of which produced a blue color. The absorbance of the solution was read by the spectrophotometer at a wavelength of 765 nm.

This standardization was performed in a 10 ml volumetric flask. In this way, the following materials were added to the 0.5 ml of each concentration extract (10, 12.5, 25, 50, 100, and 200μg/ml) 95% ethanol (1.5 ml), 10% aluminum chloride solution (0.1 ml), and 1 molar potassium acetate (0.1 ml) and distilled water (8.2 ml). After mixing, this was keptat room temperature for 30 min and finally, the absorbance of the solution for each sample was read at 765 nm and flavonoid content was measured by quercetin standard curve.

### DM preparation

DM with a purity of > 99% was prepared (Sigma-Aldrich Co., Germany) and dissolved in corn oil. This solution was fed to mice with the dose of 0.6 mg/kg.bw (1/10 LD50) by gavage (1).

### Animals

In this experimental study, 35 adult male mice (25–30 gr) were used. Also, animals were kept in the standard conditions of temperature and humidity. They had free access to food and water. Then, they were randomly divided into five groups (*n* = 7/each) and fed for 28 consecutive days via gavage. Group 1 (Control) received only similar volume of normal saline. Group 2 received 0.2 ml corn oil. Group 3 (GT) received only GT of 150 mg/kg.bw. Group 4 (DM) received the DM at a dose of 0.6 mg/kg.bw (1/10 LD50) in corn oil. Group 5 (GT + DM) received both GT (150 mg/kg.bw) and DM (0.6 mg/kg.bw) (5, 12). At the end, animals were sacrificed by spinal dislocation and their testes were removed from the abdominal cavity. One of the testicles was quickly frozen in liquid nitrogen for biochemical tests, and the opposite side was fixed in formalin buffer 10% for histological and immunohistochemistry evaluation. The blood was collected from the animal's heart and the serum was separated and stored at -20°C for the evaluation of testosterone level.

### Sperm count

After the separation of the epididymis, the sperms were extracted from its tail part and transferred to 2 ml of culture medium (Hepes buffered Ham's F10) and incubated for 5 min at 37°C. Subsequently, sperm count was performed in accordance with the WHO protocol using a hem cytometer.

### Sperm motility

10 μl of sperm suspension prepared in the culture medium was placed on a microscope slide and immediately examined in 10 fields using an optical microscope (magnification ×400).

### Sperm morphology 

In this way, 10 μl of sperm suspension was mixed with 10 μl of 2% eosin. After 1 min, 200 sperm from each animal were examined using an optical microscope (magnification ×400).The spermatozoa with abnormal head or tail were counted. The sperm with a large or small head, abnormal head shape or with two heads, separated head from the tail, as well as short, long, double, multiple, broken, hairpin, bent tails were reported as abnormal spermatozoa and expressed as percentages.

### Histological evaluation

For histological studies, six testes from each group was immediately fixed in 10% buffered formalin (Sigma, UK) solution after the removal for 24 hr. The fixed tissues were dehydrated and embedded in the paraffin. Next, the 5-micron sections were prepared and hematoxylin-eosin (H&E) staining was performed. From each testis, 100 seminiferous tubules were evaluated using an optical microscopy (magnification ×400). The percentage of seminiferous tubules with complete spermatogenesis was assessed. Johnson's score was carried out to study the maturity and quality of seminiferous tubules. The tubules were rate 1 to 10 based on the following criteria:

• The atrophic tubules were defined as seminiferous tubules with no epithelial (neither germ cells nor Sertoli cells).
• There were no germinal cells and only Sertoli cells were recognized.
• Only spermatogonia were presented.
• No spermatozoa or spermatid were observed. Only a small amount of spermatocytes were seen.
• No spermatozoa or spermatids were seen. A large amount of primary spermatocyte was recognized.
• No spermatozoa or spermatids pulp was seen, but a few primary round spermatids were presented.
• No adult spermatozoa and spermatid were seen, but a large number of primary spermatids was seen.
• Less than five spermatozoa were seen in each tube, and few mature spermatids were seen.
• There were a large number of mature spermatids, but the epithelium was degraded and the rounded and regular lumen was not seen.
• Complete spermatogenesis and perfect tubules with the presence of a large number of spermatozoa was located on the round, regular lumen were seen (13).

### Histomorphometric evaluations of testicular tissue

For quantitative evaluations of the seminiferous tubules parameters, an optical microscope with the software of OLYSIA, GmbH 3.2 (Japan) was used. From each animal, 100 seminiferous tubules with round or almost rounded sections were randomly selected and determined. The tubules with oblique sections were not studied. The height of the epithelium was calculated from the distance from the basement membrane of the germinal epithelium to the lumen of the tubule in micrometer an optical microscopy (magnification ×100). The diameter of the tubule was also measured at a distance between two similar points of the basement membrane (opposite sides) in micrometer.

### Biochemical evaluation

Malondialdehyde (MDA) as a final product of lipid peroxidation is a compound that has been used as an indicator of oxidative stress. This compound reacts with 2-thiobarbituric acid reactive substances and generates a red adduct. To evaluate the level of MDA, the samples were taken from testes frozen at -80°C. The absorbance of the samples was measured using spectrophotometry. MDA data were calculated in micromole per microgram of protein.

### Assessment of testosterone level in serum

In this study, a testosterone kit was prepared from Biotech Company (Cat.NO: E0260M1) to measure the serum levels of testosterone.

### Immunohistochemistry (IHC) evaluations

To perform the IHC-based studies, sections obtained from testicular tissue, the tissue was prepared and incubated in a normal serum for blocking non-specific regions. The sections were then incubated in the Caspase-3 polyclonal antibody of rabbit (Abcam Co., Cat.NO: GR224831-2,UK) in PBS solution overnight at 4°C. The next day, sections were washed in PBS and incubated with a horseradish peroxidase conjugated secondary antibody for (Abcam Co., Cat.NO:GR2623314-4, UK) 2 hr, and subsequently stained using DAB for 5 min. Finally, after dehydration, the coverslip was glued to the slides using cyanoacrylate cement. For negative control, the primary antibody was removed. In order to access the quantitative factors of tissue (percentage of total reaction), five photos were taken from each sample using an optical microscope and MacBiophotonics Image J 1.41 software. The results were expressed as the percentage of positive reaction in tissues of different groups (13).

### Ethical consideration

This study was managed at the Mazandaran University of Medical Sciences, Sari, Iran. The experimental standards were accepted by the Institutional Animal Care and Use Committee. The animals were provided by the Laboratory Animal Research Center of Mazandaran University of Medical Sciences and approved by the ethics committee (IR.MAZUMS.REC.1395.2622).

### Statistical analysis

Data were analyzed using Graph Pad Prism software (6.07) and analyzed by one-way ANOVA and Tukey's post-hoc test. p < 0.05 was considered as a significant level.

## 3. Results

### HPLC characterization of the GT extract

The amount of water-soluble extractive was 10.48%, total phenol as based on gallic acid and total flavonoid as based on quercetin were 26.82 ± 0.085% and 4.088 ± 0.208%, respectively.

### Sperm parameters findings

In the present study, the comparison of sperm count, motility, and morphology abnormalityin the Control, and Oil groups have no significant differences. The sperm count in the group exposed to DM decreased significantly compared to the Control group (p ≤ 0.0001, Table I). Receiving of GT in DM treated mice significantly increased the concentration of spermatozoa in this group (65.8 × 10${}^{6}$) compared to the DM group (56.6 × 10${}^{6}$) (p = 0.03). However, the sperm count in the group receiving GT + DM was also significantly lower than the control group (79.5 × 10${}^{6}$, p ≤ 0.001).

As shown in Table I, the sperm motility in the DM group was significantly reduced compared to the control, oil, and the GT groups (p ≤ 0.0001). The receipt of GT significantly increased the sperm motility in the GT + DM group as compared to the DM group (p ≤ 0.001). Nevertheless, the administration of the GT did not improve the sperm motility in the animals receiving the GT as well as the control group. In other words, the GT was able to neutralize the effect of the DM, but not completely. Therefore, there was a significant difference between GT + DM and control groups (p ≤ 0.0001). Furthermore, Table I also shows the role of DM in increasing morphological abnormalities of sperm. In the group receiving DM, the morphological abnormalities of sperm increased significantly compared to the control groups (p ≤ 0.0001). GT in the group of GT + DM was able to reduce the morphological abnormalities of sperm in comparison with the DM group (p ≤ 0.0001, Table I).

### Histological findings

**Figure 1:**
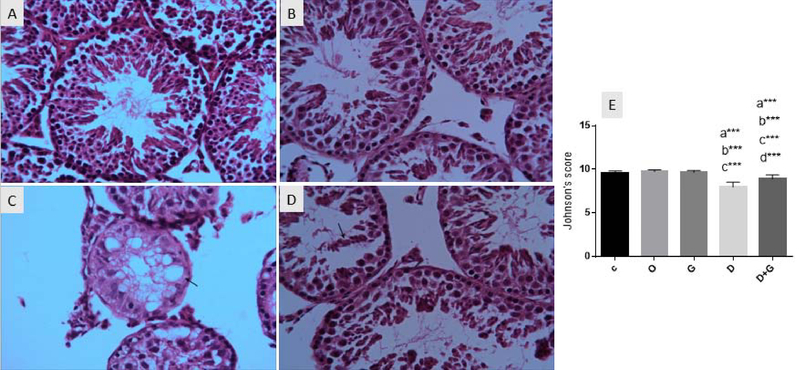
Photomicrograph of mouse testicles stained with H & E (magnification: ×40): (A) Normal testicular tissue structure of control mice. (B) Irregular seminiferous tubules and destroyed epithelium into the lumen (arrow) in the mice that received DM. (C) Destruction of spermatozoa and vocualized cells (arrow) in DM group mice. (D) Seminiferous tubules have a slight loss of epithelium and improved spermatogenesis in GT + DM group is seen. (E)Comparison of Johnson's score results in different groups. Values are expressed as Mean ± SD. a: Significant difference compared to the Con group (p < 0.001) b: Significant difference compared to the oil group (p < 0.001) c: Significant difference compared to the GT group (p < 0.001) d: Significant difference compared to the DM group (p < 0.001) **: p < 0.001       C: Control       O: Oil       G: Green tea       D: Deltamethrin

The results of the histopathological examination are shown in Figure 1. The control groups have normal tissue structure. Some pathological features such as folding the most of seminiferous tubules, multinuclear cells, vacualized cell, destruction of both Sertoli cells and germ cells, destruction of spermatozoa and germinal epithelium in some of the testicular tissue sections of the DM group were reported. These pathological changes reduced the spermatogenesis in the seminal tubules of the DM-receiving mice, evaluated using Johnson's score. The mean Johnson's score of DM group was significantly reduced compared to the control group (p ≤ 0.0001). In the GT + DM group, GT could improve the histopathologic alterations induced by DM in the testicular tissue and statistically increased the mean Johnson's score in comparison with the DM group (p ≤ 0.0001).

### Histomorphometric findings of the testes (epithelial height of the seminiferous tubules)

**Figure 2:**
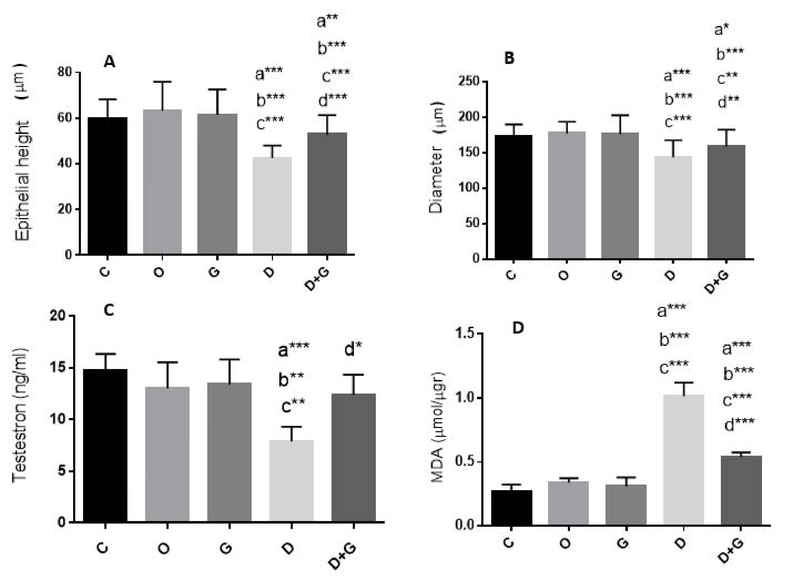
Comparison of the diameter and epithelial height of the seminiferous tubules of the testis, testosterone, and MDA levels in different groups. Values areexpressed as Mean ± SD. a: Significant difference compared to the con group (p < 0.05) b: Significant difference compared to the oil group (p < 0.001) c: Significant difference compared to the GT group (p < 0.01) d: Significant difference compared to the DM group (p < 0.01) : p < 0.05       **: p < 0.01       ***: p < 0.001 C: Control       O: Oil       G: Green tea       D: Deltamethrin

As shown in Figure 2(A), the height of the epithelium in the seminiferous tubules of the mice exposed to DM was significantly reduced compared to the control animals (p ≤ 0.0001). GT in the GT + DM exposed group had the toxicity-eliminating effects and significantly increased the height of epithelium compared to the DM group (p ≤ 0.0001, Figure 2 (A)). However, this increase in the height of the epithelium in the GT + DM group (52.8 ± 5.8) was not as well as a control group (59.6 ± 8.7). Thus, the extract could reduce the toxic effects of DM; but not completely. Therefore, there was a significant difference in the height of the epithelial cells between the groups of DM + GT and the control groups (p = 0.004). The diameter of the seminiferous tubules in the DM group was significantly decreased compared with the control group (p ≤ 0.0001, Figure 2 (B)). The diameter of the seminiferous tubules in the DM + GT group (159.3 ± 23.1) was significantly increased compared with the DM group (143.7 ± 23.7, p = 0.003). This indicates the protective effect of GT against toxicity induced by DM. However, this increase in diameter was not at the normal size. So, the diameter of seminiferous tubules in the DM + GT group was still significantly lower than the control group (p = 0.02).

### Testosterone levels 

The oral administration of DM for 28 days significantly reduced blood testosterone level compared to the control groups (p = 0.0003, Figure 2 (C)). The levels of testosterone in animals administered GT + DM were significantly increased in comparison with the DM group (p = 0.01). The GT in this dose elevated the levels of testosterone to the normal levels recorded for control groups.

### Biochemical findings

Malondialdehyde level have been shown in Figure 2 (D) for all experimental groups. In the present study, the comparison of MDA produced in testis tissue between control groups did not show any significant difference. The oral administration of DM in mice resulted in a significant increase (1.01 ± 1.0) in the lipid peroxidation compared to the control group (0.27 ± 0.05) mice (p ≤ 0.0001). In the group that received the GT extract and DM, there was a significant decrease (0.33 ± 0.03) in the MDA (peroxidation lipid) levels compared to the DM group (p ≤ 0.0001), which indicated that the GT reduced lipid peroxidation. Although the lipid peroxidation decreased in the GT + DM group, it was still significantly higher than the control group (p ≤ 0.0001). In other words, GT in this dose has been able to reduce the toxic effect of DM on testicular tissue but did not completely neutralize it.

### Immunohistochemistry (IHC) findings

**Figure 3:**
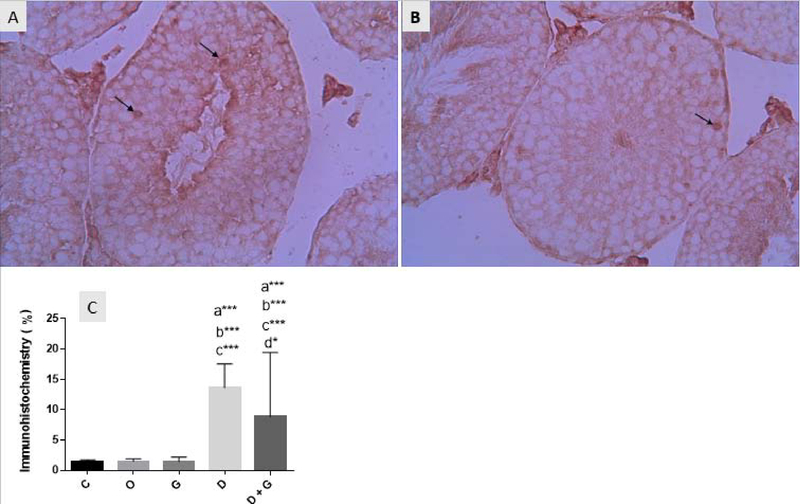
Immunohistochemical photomicrograph of mouse testis (magnification: ×40): (A). The DM group represents the expression of caspase-3 protein which displays brown in epithelial regions (arrow) (B). In the group of DM+GT, GT reduced the expression of caspase-3 in mice exposed to DM and improved the density of nuclei (C). Comparison of the mean expression of caspase-3 in the testicles of different groups (*n* = 5). a: Significant difference compared to the con group (p < 0.001)       b: Significant difference compared to the oil group (p < 0.001) c: Significant difference compared to the GT group(p < 0.001)       d: Significant difference compared to the DM group (p < 0.05) : p < 0.05       ***: p < 0.001      C: Control      O: Oil       G: Green tea       D: Deltamethrin

In IHC evaluations, the caspase-3 positive cells display brown color. Immunohistochemistry of the testicles showed the normal features with normal seminiferous tubules and interstitial tissue structures in the control and the GT groups. In the DM group, the activity of caspase-3 in spermatogonia was confirmed to be moderate and in primary spermatocytes and spermatids, it was weak. In other words, exposure to DM (3.93 ± 6.62) significantly increased the expression of caspase-3 compared to the control groups (p ≤ 0.0001), while the GT in the DM + GT group (8.87 ± 10.58) improved the alterations in the seminiferous tubules and significantly decreased the number of caspase-3positive cells in comparison with the DM group (p = 0.03; Figure 3).

**Table 1:** Comparison of the count, motility, and morphology abnormality of spermatozoa in different groups (n = 5)


**Groups**	**C**	**O**	**GT**	**DM**	**DM+GT**
Count (×10${}^{6}$ ML)	79.5 ± 3.4	75.8 ± 4.5	74 ± 6.3	a*** 56.6 ± 4.6	a**b* 65.8 ± 3.7
Motility (%)	35.1 ± 3.2	34.7 ± 3.3	33 ± 2	a*** 17 ± 1.5	a***b** 23.8 ± 0.49
Abnormal Morphology (%)	14.3 ± 3.3	17.4 ± 1.4	18.4 ± 2.3	a*** 35.2 ± 5.2	b*** 20.5 ± 3.4
Note: Values are expressed as Mean ± SD					
a: Significant difference compared to the Control group; b: Significant difference compared to the DM group					
*: p < 0.05 **: p < 0.01 ***: p < 0.001 C: Control group					
O: Oil GT: Green tea extract group DM: Deltamethrin groups					

## 4. Discussion

Because of the widespread use of pesticides in domestic and industrial sectors and their toxic effects, these bio-contaminants may reduce the reproductive performance of men (12). In the present study, DM at the dose of 0.6 mg/kg causes increased lipid peroxidation, spermiotoxicity, apoptosis, and changes in the mouse testicular structure. GT administration reduced MDA. Moreover, GT improved sperm parameters in the DM-treated mice. Also, GT decreased apoptosis and changes in the testicular tissue.

The results of this study, with respect to DM toxic effect on spermatozoa parameters, indicated that DM decreased the count and motility of spermatozoa and increased their morphological abnormalities. Slima *et al*. orally exposed mice to DM daily for 35 consecutive days. Their results presented significant decreases in the count, motility, and vitality of spermatozoa in mice (14). Oda and colleagues determined the adverse effects of DM on sex organs and fertility of male rats. Then, they evaluated the protective roles of the Vitamin E and selenium combination against the harmful effects of DM on fertility. They showed the count, motility, and vitality of epididymal spermatozoa, as well as serum testosterone levels reduced in mice that only received DM (4). DM inhibits sperm motility by diminishing the content of ATP. Additionally, the deficiency of ATP leads to sperm abnormalities that subsequently decrease fertility (15). On the other hand, the induction of oxidative stress and the peroxidation of membrane fatty acids lead to the loss of fluidity of the membrane and ion channel, resulting in a gradual loss of motility and the ability of the spermatozoa to attach to oocyte (16). Our results revealed that the levels of serum testosterone significantly decreased in mice exposed to DM. Reducing the activity of dehydrogenase is probably due to decreased testosterone secretion related to impaired steroid genesis as oxidative outcomes (17). Moreover, it can be expressed that the decreased level of hormones is associated with the direct effect of toxic substances on the pathway of biosynthesis of androgens in the gonads and additionally indirect effects on the hypothalamus-anterior pituitary gland, which subsequently may affect the testicular and sexual function (18). Therefore, reducing testosterone may lead to diminished spermatozoa count and mobility, as well as testicular abnormalities in mice exposed to DM (19). Likewise, previous studies have shown that pesticides may interfere with the mitochondria membrane of the interstitial (Leydig) cells and disrupt the testosterone biosynthesis by reducing the transmission of cholesterol to mitochondria and its conversion to pregnenolone, thus resulting in reduced production of testosterone in the testis (20). The results of this study showed that the mean Johnson's score in mice exposed to DM was lower than the control groups. DM caused some histological changes in the testicles, including degenerated seminiferous tubules with incomplete spermatogenesis, decreased spermatogenic cells, and—in some cases—complete destruction of spermatogenic cells, vacualized cells, and degeneration of germinal cells. Our results confirm the study of Rashid and co-worker that showed similar histological changes in the testis of DM-exposed mice (21).

The morphometric results in the present study showed a decrease in the diameter of the seminiferous tubules and the height of the epithelium in the DM-received groups compared to the control groups. In a similar study, Kilian *et al* evaluated the low concentrations of DM in competition with several other bio-pollutants on sex parameters in Spraque-Dawley male rats. They observed that the height of epithelial and similarly the diameter of the seminiferous tubules in the treated groups decreased compared to the control group (22). Reducing the height of the epithelium in the seminiferous tubules can be due to the separation of these cells from the basal lamina, which leads to vascular insufficiency and, consequently resulting in sloughing these cells into the lumen, which causes a reduction of epithelial height in the DM group (23). It is also probable that the reduction of the epithelial height of the seminiferous tubules exposed to DM is due to the inhibition of mitotic divisions in Type B Spermatogonia and the prolongation of the G1 phase of the cell cycle (24) and/or due to endocrine activity, it may be disrupted (22). Consequently, the reduced height of epithelium leads to a defect in the production of spermatozoa that causes oligospermia or azoospermia types of infertility (25). It has been suggested that reducing the diameter of the seminiferous tubules can be due to the degeneration of the germ cells and their reduction (23). The researchers considered that this decrease may be associated with endocrine dysfunction as a result of DM effects (23).

Apoptosis is the key mechanism of degenerative diseases that are caused by some factors such as toxins (26). Immunohistochemical evaluations of our study presented that the exposure to DM significantly increased the expression of caspase-3, which plays an important role in the production of inflammatory mediators and testicular apoptosis (27). Although DM can cause apoptosis in testicular tissue, its mechanisms of action have not been investigated yet. Nevertheless, there are several suggested mechanisms to define the adverse effects of DM in the induction of apoptosis (6). In 1996, it was shown that DM caused apoptosis and cell death of thymocytes through the binding of calcium-calmodulin to protein kinase-phosphate in mice model (28). DM can similarly activate calcium-bindingendonuclease, leading to DNA fragmentation, which is a necessary step in the process of apoptosis (6). The results of biochemical studies in our study demonstrated an increase in MDA of testis tissue in DM group compared to the control group. It proves the toxicity effect of DM on testicular tissue and spermatogenesis. These results are similar to the results of El-Gohary and colleagues, which indicated that DMinduced lipid peroxidation and production of nitric oxide in the plasma of mice (6). According to the reports, since DM toxicity is associated with free radicals, it is said that DM induces oxidative stress and so toxicity in mice testicles (1, 29). Therefore, the usage of antioxidants to counteract the production of free oxygen radicals is considered an important principle in decreasing this type of toxicity (1). Green tea is one of the fairly common drinks throughout the world (30), which has two important bioactive components: polyphenols and flavonoids. There are four main polyphenols in the tea extract classified as catechins: Epicatechin, epigallocatechin, epicatechin gallat, and epigallocatechin gallate (31). Our findings indicated that the simultaneous use of GT with DM in experimental mice increased the count and motility of spermatozoa, the levels of serum testosterone, the mean Johnson's score, and the morphometric measurements such as the height of the epithelium and the diameter of the seminiferous tubules and improved the morphological abnormalities and spermatozoa quality in the group that received GT + DM in comparison with those receiving DM. On the other hand, GT could reduce the degenerative changes in the testicles and also the expression of Caspase-3 (apoptosis) and MDA levels in GT + DM-exposed mice compared to those treated with DM. Sato and co-worker in a study demonstrated the protective effects of GT against doxorubicin-induced spermatogenesis in mice. They also revealed that GT has aromatase inhibitor activity, which could be the main cause of improved serum levels of testosterone (10). According to a study by researchers, the effect of GT in reducing apoptosis is related to its antioxidant activity of catechins (32). Abolfathi in a study revealed that GT has improved the antioxidant status of the liver tissue in diabetic rats exposed to streptozotocin (33). The precise mechanisms involved in the beneficial effects of GT on spermatogenesis are still unclear (34, 35). The main mechanism of this drink is related to its antioxidant properties and mainly catechins, which protects against the toxicity induced by oxidative stress in the organs such as testicles (34). Catechins can strongly eliminate free radicals, specially the most active radical hydroxyl that initiates lipid peroxidation, and so improve lipid peroxidation due to their unpaid electron (15). In addition, mineral content of tea such as zinc, selenium, and manganese act as a cofactor in the efficiency of oxidative enzymes. Polyphenols affect directly as an antioxidant and they also reduce oxidation levels by indirect mechanisms (9, 15). On the other hand, the improvement in the sperm parameters in DM+GT group may also be due to the increased testosterone levels. The testosterone and gonadotropins like FSH and LH are essential indicators in male fertility and also require to maintain spermatogenesis (36). Consequently, increasein the levels of serum testosterone, the mean Johnson's score, the morphometric measurements, and the reduction in the degenerative changes in the testicles, apoptosis, and MDA levels in mice exposed to DM+GT are related to GT antioxidant activity of catechins (11, 15, 32). However, the molecular targets and the exact mechanism of action of the polyphenols present in GT in the inhibition of apoptosis are still unknown, and further studies are needed (37). The limitation of this study was evaluation of other oxidative stress markers such as GSH, ROS, PC of GT.

## 5. Conclusion

According to the findings of this study, we concluded that DM causes testicular toxicity, but GT as an antioxidant agent improved testicular injury. Also, GT with anti-apoptotic property decreased apoptosis by reducing the expression of caspase-3.

##  Conflict of Interest

There is no conflict of interest in this study and publication.
